# Renal Allograft Biopsies with Polyomavirus BK Nephropathy: Turin Transplant Center, 2015–19

**DOI:** 10.3390/v12091047

**Published:** 2020-09-20

**Authors:** Elisa Zanotto, Anna Allesina, Antonella Barreca, Francesca Sidoti, Ester Gallo, Paolo Bottino, Marco Iannaccone, Gabriele Bianco, Luigi Biancone, Rossana Cavallo, Cristina Costa

**Affiliations:** 1 Microbiology and Virology Unit, University Hospital City of Health and Science of Turin, Corso Bramante 88, 10126 Turin, Italy; francesca.sidoti@unito.it (F.S.); paolo.bottino@unito.it (P.B.); marco.iannaccone@unito.it (M.I.); gabriele.bianco@unito.it (G.B.); rossana.cavallo@unito.it (R.C.); cristina.costa@unito.it (C.C.); 2 Nephrology Unit, University Hospital City of Health and Science of Turin, Corso Bramante 88, 10126 Turin, Italy; anna.allesina@unito.it (A.A.); egallo@cittadellasalute.to.it (E.G.); luigi.biancone@unito.it (L.B.); 3 Pathology Unit, University Hospital City of Health and Science of Turin, Corso Bramante 88, 10126 Turin, Italy; antonella.barreca@unito.it

**Keywords:** BKV, nephropathy, immunosuppression

## Abstract

Background: In kidney transplant patients, polyomavirus-associated nephropathy (PVAN) represents a serious complication; the key factor for the development of PVAN is immunosuppression level and modulation of anti-rejection treatment represents the first line of intervention. Allograft biopsy and histology remain the criterion standard for diagnosing PVAN. Methods: All consecutive renal biopsies with the diagnosis of PVAN carried out at the University Hospital City of Health and Science of Turin over a five-years period were studied. Renal allograft biopsy was performed due to renal function alterations associated to medium-high polyomavirus BK (BKV)-DNA levels on plasma specimen. Results: A total of 21 patients underwent a first biopsy to diagnose a possible BKV nephropathy, in 18, a second biopsy was made, in eight, a third biopsy, and finally, three underwent the fourth renal biopsy; following the results of each biopsies, immunosuppressant agents dosages were modified in order to reduce the effect of PVAN. Conclusions: In this study, the clinical and histological features of 21 kidney transplant recipients with BKV reactivation and development of PVAN are described. To date, the only treatment for PVAN consists in the reduction of immunosuppressive agents, constantly monitoring viral load.

## 1. Introduction

Polyomavirus BK (BKV) belongs to the Polyomaviridae family and is ubiquitously distributed throughout the general population [1,2]. Following primary infection that usually occurs early in the childhood via the respiratory tract and is mainly asymptomatic, BKV remains latent at different sites, including the renourinary tract [3,4]. From latency sites reactivation may occur, particularly in the presence of abnormal immune conditions, such as transplant recipients [5]. 

In transplant patients, two serious complications associated to BKV reactivation have been reported: polyomavirus associated nephropathy (PVAN) and polyomavirus associated hemorrhagic cystitis (PyVHC) [6,7,8]. Approximately 30–50% of kidney transplant patients with high-level of BKV viruria and viremia progress to PVAN [9,10,11,12]. Determinants for PVAN include recipient characteristics [7] such as older age, male gender, and low or absent BKV-specific T-cell activity; posttransplant factors such as acute rejection and antirejection treatment, steroid exposure, higher immunosuppressive drug levels, tacrolimus-combinations compared to cyclosporine or mTOR inhibitor-combinations, as well as viral features, such as BKV genotype [13,14,15,16,17]. The immunosuppression level represents the key factor for the development of PVAN and modulation of anti-rejection treatment is considered the first line of intervention [18].

Patients under immunosuppressive treatment should be monitored for BKV reactivation by evaluation of viral load on plasma and/or urine specimens, particularly in the first year after transplantation. 

In this study, we describe the features of PVAN cases observed at the Turin Renal Transplant Center (Piemonte Region, North Western Italy) over a period of five years. 

## 2. Materials and Methods

This descriptive study has been conducted at the University Hospital City of Health and Science of Turin, in particular with the participation of Microbiology and Virology, Pathology and Nephrology Units referring to the Piemonte Region Renal Transplant Center. The Transplant Center includes three different sites, in particular two located at the University Hospital City of Health and Science of Turin (Molinette Presidium for adults and Regina Margherita Children Hospital for pediatric transplantation) and one at the Maggiore Hospital of Charity in Novara. The Piemonte Region Renal Transplant Center is the first in Italy for activity volume, in particular at the Molinette Presidium approximately 100–150 kidney transplants (single and double) are performed every year.

According to the Transplant Center practice, following transplantation, all patients are discharged with basic therapy (with slight changes customized for each subject) consisting of anti-thymoglobuline (ATG), Basiliximab (BAS), Tacrolimus (TAC), mycophenolate mofetil/mycophenolate (MMF/MPA), and steroids (St); in the occurrence of clinical events or based on the characteristics of the patient and the type of intervention, some modifications in this treatment can be made [19], as reported in Table 1.

All patients are monitored for renal function and BKV reactivation. In particular, viral load is monitored by evaluation of BKV DNA on plasma samples twice monthly in the first three months post-transplantation, then every three months; in the presence of renal function abnormalities or on the basis of clinical judgement, intervals of monitoring are reduced to twice monthly.

Persistently high levels of BKV DNA in plasma (i.e., >4 log10 copies/mL for more than 4 weeks) are considered for a presumptive diagnosis of PVAN in kidney transplant patients [20]; however, the definitive diagnosis is made by histological evaluation of renal allograft biopsy, confirmed by immunohistochemistry or in situ hybridization [4,13]. In the occurrence of high levels of BKV DNA on plasma specimens followed by renal function abnormalities, biopsy is performed for evaluation and staging of PVAN. All consecutive biopsies with the diagnosis of PVAN carried out at the University Hospital City of Health and Science of Turin over a five-years period (January 2015–December 2019) have been evaluated in this study.

For BKV DNA quantitation, extraction was carried out with the fully automated instrument QIAsymphony^®^ SP/AS, using the kit “QIAsymphony^®^ DSP Virus/Pathogen Midi kit” and following the manufacturer instructions (extraction protocol “Virus Cell free 500_V3_DSP_default IC”; plasma volume for extraction, 500 µL; elution volume, 85 µL)(Qiagen, Milan, Italy). Nucleic acid amplification was performed with BKV ELITe MGB^®^ Kit, (ELITech Group, Turin, Italy) on 7500 Fast Dx Real-Time PCR Instrument with the following thermal profile: decontamination 50 °C 2 min, initial denaturation 94 °C 2 min, amplification and detection (45 cycles) 94 °C 10 s, 60 °C (fluorescence acquisition) 30 s, 72 °C 20 s, dissociation (optional) 95 °C 15 s, 40 °C 30 s, 80 °C 15 s. The probe with ELITe MGB^®^ technology specific for BKV marked with the FAM fluorophore is activated when hybridized with the specific product of the amplification reaction for BKV. The probe with ELITe MGB^®^ technology specific for Internal Control labeled with AP525 fluorophore (equivalent to VIC) is activated when hybridized with the product of the amplification reaction for the Internal Control.

For histological evaluation, renal biopsies were fixed in Serra fluid, paraffin embedded, and stained with periodic acid-Schiff reagent, Masson trichrome, phosphotungstic acid hematoxylin (PTAH), and acid fuchsin orange G (AFOG). Immunohistochemistry with immunoperoxidase staining was performed on fixed material using mouse monoclonal anti-SV40 antibody (pre-diluted clone MRQ-4 Roche) and Rabbit monoclonal C4d (prediluted clone SP91 (Roche, Monza, Italy).

## 3. Results

Over the study period, PVAN was diagnosed in 21 kidney transplant patients (male/female: 17 (80.9%)/4 (19.1%)). Table 2 shows demographic features, underlying disease and therapy at discharge of the study subjects; in Table 3 results for renal biopsy and concomitant BKV DNA plasma levels are reported.

Overall, one patient presented no evidence of nephropathy at the first biopsy, 3/21 patients (14.3%) had grade A nephropathy, 11/21 (52.4%) B1, 4/21 (19%) B2, 1/21 (4.7%) B3, and 1/21 (4.7%) C, respectively. All patients presented interstitial fibrosis and mild to moderate tubulitis, often associated with capillaritis and infiltration of inflammatory cells. A mild acute glomerulopathy was observed only in one patient; three subjects exhibited acute rejection, and a further three patients developed mild acute calcineurin inhibitor toxicity. Immunohistochemical investigation of material fixed with anti-C4d antibodies resulted positive in three patients, in the tubular and glomerular basement membrane, as well as in the interstitial capillaries.

Following the results of the biopsies, immunosuppressant agents dosages were modified in order to reduce the effect of PVAN, in particular a common initial approach consisted into the reduction of the calcineurin inhibitor dose by 25% to 50% with target tacrolimus trough levels <6 ng/mL or cyclosporine levels <150 ng/mL. This was followed by a dose reduction of the antiproliferative agent by 50% followed by its discontinuation, depending on the response [20]. All the details of changes in therapy after the first biopsy are reported in Table 4.

All patients were then monitored again to observe the progress of BKV DNAemia following changes in immunosuppressive treatment; in 18 patients a second biopsy was performed (Table 5).

Signs of PVAN were not observed in 7 of 18 patients (38.9%) who underwent the second biopsy; however, levels of interstitial fibrosis were increased in comparison to the first biopsy (83.3% moderate/severe cases in the second biopsy and 28.6% of moderate cases in the first biopsy). Capillaritis, tubulitis and inflammatory infiltrate presented a slight deterioration in comparison to the first biopsy; viral load decreased by 1–3 Log_10_ in 55.5% of cases, remained stable in 33.3%, and increased in 11.1% of cases.

The dosages of immunosuppressive therapies were further modified, in particular the levels of tacrolimus were reduced (up to 1–2 ng/mL) as well as the dosages of everolimus (3–4 ng/mL); steroid levels were decreased by up to 2.5 mg.

A third renal biopsy was required for eight patients, the results of which are shown in Table 6. With the exception of two patients with extremely high BK viral loads (6 Log10), BKV-DNA level reduced in six of these eight subjects, four patients presented no further signs of PVAN at the biopsy, two developed grade B2 and two grade C nephropathy, respectively. For all subjects the fibrosis picture aggravated up to severe levels, only one patient evidenced glomerulopathy and another shows signs of chronic rejection.

No relevant modification in current treatment was made. 

In consideration of persistently high levels of BKV-DNA in plasma specimens, a fourth biopsy was performed in three patients (Table 7). In patient #1 severe global sclerotic evolution, moderate vascular damage and pictures referable to PVAN were not further observed. Patients #18 and #19 still exhibited aspects of PVAN with moderate fibrotic damage and inflammatory infiltrate, in particular, patient # 18 presented severe vascular damage mainly of the arteriolosclerosis type and patient #19 developed acute transplant glomerulopathy with associated chronic glomerulopathy. For patients #1, 7, 8, 11, and 19, renal damage due to BKV replication and therefore to nephropathy led to loss of renal function, despite the reduction of immunosuppressive therapy. For these five patients it was necessary to return to hemodialysis, patient # 7 was again placed on the waiting lists for a new kidney transplant and patient #19 underwent allograft removal.

**Figure 1:**
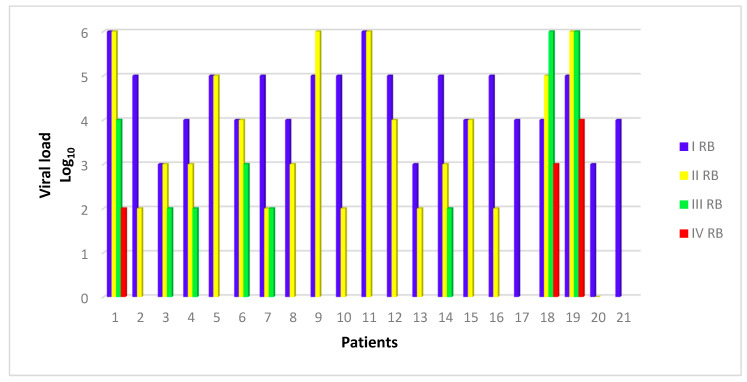
Renal biopsy (RB) and concomitant viral load (expressed as Log_10_ copies/mL). Number of biopsies for each patient: first biopsy in 21 patients, second in 18, third in eight and fourth in three.

In Figure 1, viral load of BKV expressed in Log_10_ (copies/mL) at the moment of the different biopsies in study patients is shown. 

**Figure 2:**
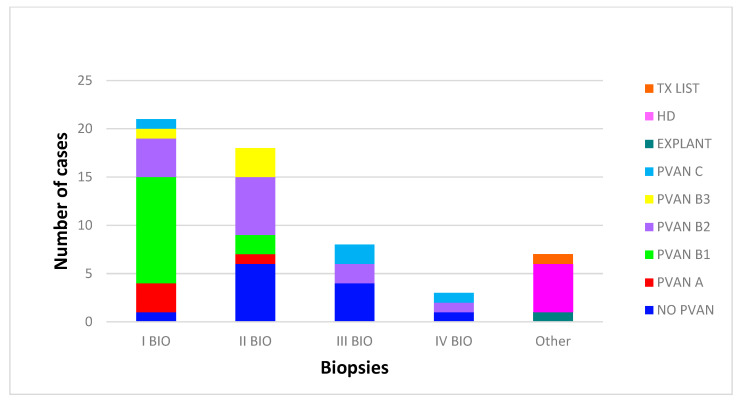
Histological results and polyomavirus nephropathy (PVAN) staging at renal biopsies. Tx list, entry in transplant list; HD, hemodialysis.

In Figure 2, the occurrence of different results/outcomes at each biopsy is reported.

## 4. Discussion

Polyomavirus-associated nephropathy is a relevant problem following kidney transplantation. In this study, the clinical and histological features of 21 kidney transplant recipients with BKV reactivation and development of PVAN have been described.

To date, the only treatment for PVAN consists into the reduction of immunosuppressive agents [19], constantly monitoring the patient for the progress of the viral load in the blood. In this study, following reduction and/or withdrawal of immunosuppressive drugs, an improvement was observed for 16 patients (76.2%), in particular as regards mycophenolate mofetil, tacrolimus, and steroids; in the remaining five patients (23.8%) the transplanted organ presented a severe damage with development of renal function abnormalities that conducted to return to hemodialysis. These findings support the relevant role played by modulation of immunosuppression in the treatment of PVAN.

Several risk factors have been described for BKV replication and PVAN developing in renal transplanted recipients, and it is well known that immunosuppression plays an important role [21,22]; in fact, PVAN started to emerge as a relevant complication of renal transplantation towards the mid-1990s when triple immunosuppressive therapy regimens were introduced [23]. The pathogenesis of PVAN probably involves multiple risk factors, which include immunosuppression and characteristics of the patient, the transplanted organ, and the virus, but intense immunosuppression is considered the major risk factor for PVAN [13,24]. The relevant role played by immunosuppression and the fact that the first line of treatment consists in the modulation of immunosuppression is supported by our findings in that more than 75% of study patients evidenced a histological improvement by adopting this strategy. Similarly, also viral load could benefit from immunomodulation, as evidenced by a reduction of at least one up to three log values of BKV load in plasma specimens.

Apart of immunosuppression, other factors may have an impact on the outcome of renal transplantation. For example, patient determinants increasing the risk for PVAN may include older age and male gender, white ethnicity, diabetes mellitus, and a negative BKV serostatus in pediatric recipients [5,25,26,27]. Moreover, organ determinants may play a role as suggested by the fact that PVAN is rare in transplant recipients of non-renal solid organs despite exposure to the same combinations and concentrations of immunosuppressive drugs [28,29]. From a virological point of view, BKV determinants include changes in immunologically relevant epitopes of the capsid protein VP-1 leading to immune evasion in some patients and sequence alterations which possibly might affect viral gene expression and/or replication, generating new viral strains with new genetic mutations [30,31,32]. Virological variance and changing virulence by altering DNA sequence needs further studies. This could be relevant considering immune response against donor strain transmitted to the recipient and responsible for PVAN.

In conclusion, in this study we describe the occurrence of PVAN in the Turin Transplant Center over the period of five years and the outcome of these cases taken into consideration the histological evaluation and the modifications in immunosuppressive treatment supporting the relevant role played by immunomodulation as therapeutic strategy.

## Figures and Tables

**Table 1:** Therapy at discharge. Anti-thymogobuline (ATG), Basiliximab (BAS), Tacrolimus (TAC), mycophenolate mofetil/mycophenolate (MMF/MPA), and steroids (ST).

Patients	Therapy at Discarge					
	ATG	BAS	TAC	MMF	ST	Eculizumab/Rituximab
**1**	8 × 25 mg	2 × 20 mg	10–12 ng/mL down to 8–10	500 mg × 2 down to 250 mg	750 mg down to 2.5 mg	
**2**	-	2 × 20 mg	10–12 ng/mL down to 8–10	500 mg × 2	750 mg down to 5 mg	
**3**	840 mg	-	10–12 ng/mL down to 8–10	500 mg × 2	750 mg down to 5 mg	
**4**	-	2 × 20 mg	10–12 ng/mL down to 8–10	500 mg × 2	750 mg down to 5 mg	
**5**	-	2 × 20 mg	10–12 ng/mL down to 8–10	360 mg × 2 (MPA)	750 mg down to 5 mg	
**6**	-	2 × 20 mg	10–12 ng/mL down to 8–10	360 mg × 2 (MPA)	750 mg down to 5 mg	900 mg down to 600 mg (Eculizumab)
**7**	-	2 × 20 mg	10–12 ng/mL down to 8–10	720 mg × 2 (MPA)	750 mg down to 5 mg	
**8**	-	2 × 20 mg	10–12 ng/mL down to 8–10	500 mg × 2	750 mg down to 5 mg	
**9**	-	2 × 20 mg	10–12 ng/mL down to 8–10	360 mg × 2 (MPA)	750 mg down to 5 mg	
**10**	-	2 × 20 mg	10–12 ng/mL down to 8–10	360 mg × 2 (MPA)	750 mg down to 5 mg	
**11**	525 mg	-	10–12 ng/mL down to 8–10	500 mg × 2	750 mg down to 5 mg	
**12**	-	2 × 20 mg	10–12 ng/mL down to 8–10	500 mg × 2	750 mg down to 5 mg	
**13**	-	2 × 20 mg	10–12 ng/mL down to 8–10	500 mg × 2	750 mg down to 5 mg	
**14**	625 mg	-	10–12 ng/mL down to 8–10	500 mg × 2	750 mg down to 5 mg	
**15**	-	2 × 20 mg	10–12 ng/mL down to 5–7	500 mg × 2	750 mg down to 5 mg	
**16**	-	2 × 20 mg	10–12 ng/mL down to 8–10	500 mg × 2	750 mg down to 5 mg	
**17**	325 mg	2 × 20 mg	10–12 ng/mL down to 8–10	500 mg × 2	750 mg down to 5 mg	
**18**	-	2 × 20 mg	10–12 ng/mL down to 8–10	360 mg × 2 (MPA)	750 mg down to 5 mg	
**19**	-	2 × 20 mg	10–12 ng/mL down to 8–10	360 mg × 2 (MPA)	750 mg down to 5 mg	650 mg × 2 (Riruximab)
**20**	240 mg	-	10–12 ng/mL down to 8–10	500 mg × 2	750 mg down to 5 mg	
**21**	150 g	-	10–12 ng/mL down to 8–10	360 mg × 2 (MPA)	750 mg down to 5 mg	

**Table 2:** Demographic, clinical, and therapeutic features of study patients. Anti-thymogobuline (ATG), Basiliximab (BAS), Tacrolimus (TAC), mycophenolate mofetil (MMF), mycophenolate (MPA), steroids (st), eculizumab (eculiz), rituximab (ritux), plasmapheresis (pl).

Patients	Sex	Age at TX	Disease	Therapy at Discharge	Creatinine mg/dL
1	F	60.4	Chronic renal failure	ATG + BAS + TAC + MMF + ST	1.2
2	F	70.51	Glomerulonephritis with IgA deposits	BAS + TAC +MMF + ST	3.6
3	M	48.93	Chronic renal failure	ATG + TAC + MMF + ST	2.1
4	M	34.46	Glomerulonephritis with IgA deposits	BAS + TAC + MMF + ST	1.25
5	F	22.66	Chronic pyelonephritis in congenital solitary kidney,	BAS + TAC + MPA + ST	2.4
6	M	53.28	Hemolytic uremic syndrome	BAS + TAC + MMF + ST + eculiz	1.8
7	M	64.25	Nephroangiosclerosis	ATG + BAS + TAC +MMF	2.83
8	M	68.39	Polycystosis	BAS + MMF + ST	3.88
9	M	61.74	Chronic renal failure	BAS + TAC + MPA + ST	2.9
10	M	75.62	Chronic renal failure	BAS + TAC + MPA + ST	2.5
11	M	39.80	Glomerulonephritis with IgA deposits	ATG + TAC + MMF + ST	3.5
12	M	53.92	Nephroangiosclerosis	BAS + TAC+ MMF + ST	1.2
13	M	57.77	Chronic glomerulopephritis	BAS + TAC+ MMF + ST	3.6
14	M	47.08	Ischemic tubular nephropathy	ATG + TAC + MMF + ST	2
15	M	66.60	Polycystosis	BAS + TAC + MMF + ST	2.3
16	M	55.07	Autosomal dominant polycystic kidney disease	BAS + TAC + MMF + ST	1.9
17	M	49.63	Autosomal dominant polycystic kidney disease	ATG + TAC + MMF + ST	1.5
18	M	28.76	Drug nephrotoxicity	BAS + TAC + MPA + ST	0.9
19	M	82.89	Chronic renal failure	Ritux + 3 pl + BAS + TAC + MPA + ST	1.39
20	F	24.94	Reflux nephropathy	ATG + TAC + MMF + ST	0.9
21	M	58.66	Glomerulonephritis with IgA deposits	ATG + TAC + MPA + ST	1.4

**Table 3:** First biopsy.

Patients	DNAemia (Log_10_)	PVAN	Interstitial Fibrosis	Capillaritis	Tubulitis	Interstitial Inflammatory Infiltrate	C4d	Acute Glomerulopathy	Chronic Glomerulopathy	Acute Rejection	Chronic Rejection	Cytoplasmic Vacuolation of Tubular Epithelium (Acute Toxicity by Calcineurin Inhibitors)
1	6	A	Mild	mild	mild	mild	neg	-	-	-	-	-
2	5	B1	Mild	-	mild	-	neg	-	-	-	-	-
3	3	C	Moderate	mild	moderate	mild	neg	-	-	-	-	-
4	4	B1	Mild	-	mild	mild	neg	-	-	-	-	-
5	5	B1	Mild	-	mild	mild	neg	-	-	-	-	mild
6	4	B1	Mild	mild	mild	mild	neg	-	-	-	-	-
7	5	B2	Moderate	mild	moderate	mild	neg	-	-	-	-	-
8	4	B1	Mild	mild	mild	mild	neg	-	-	-	-	-
9	5	Absent	Moderate	moderate	moderate	mild	neg	-	-	-	-	-
10	5	B2	Moderate	moderate	moderate	moderate	neg	-	-	-	-	-
11	6	B3	Moderate	moderate	moderate	moderate	neg	-	-	-	-	-
12	5	B1	Mild	mild	mild	mild	pos *	-	-	-	-	-
13	3	B1	Mild	mild	mild	mild	neg	-	-	mild vascular	-	-
14	5	A	Mild	-	mild	mild	neg	-	-	-	-	-
15	4	B1	Mild	mild	moderate	mild	pos *	-	-	mild interstitial	-	-
16	5	B2	Mild	-	mild	moderate	neg	-	-	-	-	-
17	4	B2	Mild	mild	mild	mild	neg	-	-	-	-	-
18	4	B1	Mild	mild	mild	mild	neg	-	-	-	-	mild
19	5	B1	Moderate	mild	mild	mild	pos **	mild	-	-	-	-
20	3	B1	Mild	mild	moderate	moderate	neg	-	-	moderate interstitial	-	-
21	4	A	Mild	moderate	mild	mild	neg	-	-	-	-	mild

PVAN: Polyomavirus Associated Nephropathy; Neg: Negative; pos: Positive; * in the tubular basal membrane; ** in interstitial capillaries and glomerular basal membrane.

**Table 4:** Changes in therapy after first biopsy.

Patients	Changes in Therapy After First Biopsy							
	ATG	BAS	TAC	MMF	ST	Eculizumab/Everolimus	LEFLUNOMIDE	Ig
1	/	/	5–7 ng/mL	STOP	2.5 mg		20 mg	
2	/	/	4–6 ng/mL	STOP	5 mg		20 mg	25 mg
3	/	/	6–8 ng/mL	STOP	5 mg		20 mg	
4	/	/	5–7 ng/mL	STOP	5 mg		40 mg	
5	/	/	5–7 ng/mL	MPA 180 mg × 2	5 mg		20 mg	100 mg
6	/	/	5–7 ng/mL	STOP	5 mg	1200 mg down to 600 mg (eculizumab)	/	125 mg
7	/	/	4–6 ng/mL	MPA 360 mg × 2	5 mg		40 mg	140 mg
8	/	/	5–7 ng/mL	STOP	5 mg		40 mg	150 mg
9	/	/	8–10 ng/mL	STOP	5 mg			
10	/	/	5–7 ng/mL	STOP	5 mg		20 mg	
11	/	/	4–6 ng/mL	STOP	5 mg	5–6 ng/mmL (everolimus)	40 mg	
12	/	/	2–3 ng/mL	STOP	5 mg	4–5 ng/mmL (everolimus)	20 mg	
13	/	/	8 ng/mL	STOP	5 mg			150 mg
14	/	/	8 ng/mL	MPA 360 mg × 2	5 mg		20 mg	120 mg
15	/	/	2–3 ng/mL	STOP	5 mg	4–6 ng/mmL (everolimus)	20 mg	160 mg
16	/	/	8 ng/mL	STOP	5 mg		40 mg	160 mg
17	/	/	4–6 ng/mL	STOP	5 mg		20 mg	
18	/	/	2–3 ng/mL	STOP	5 mg		20 mg	40 mg
19	/	/	5 ng/mL	STOP	5 mg	5–6 ng/mmL (everolimus)	20 mg	160 mg
20	/	/	5–7 ng/mL	STOP	5 mg			90 mg
21	/	/	3–4 ng/mL	STOP	2.5 mg		20 mg	160 mg

Anti-thymogobuline (ATG); Basiliximab (BAS); Tacrolimus (TAC); mycophenolate mofetil/mycophenolate (MMF/MPA); steroids (ST); intravenous immunoglobulins (Ig).

**Table 5:** Second biopsy.

Patients	DNAemia (Log10)	PVAN	Interstitial Fibrosis	Capillaritis	Tubulitis	Interstitial Inflammatory Infiltrate	C4d	Acute Glomerulopathy	Chronic Glomerulopathy	Acute Rejection	Chronic Rejection	Cytoplasmic Vacuolation of Tubular Epithelium (Acute Toxicity by Calcineurin Inhibitors)
1	6	B2	moderate	mild	mild	moderate	neg	-	-	-	-	-
2	2	Absent	moderate	mild	mild	mild	neg	-	-	-	-	-
3	3	Absent	moderate	mild	moderate	moderate	neg	-	-	-	mild interstitial	-
4	3	B1	Mild	mild	mild	mild	neg	-	-	-	-	-
5	5	B3	moderate	-	mild	severe	neg	-	-	-	-	mild
6	4	Absent	Mild	mild	mild	mild	neg	-	-	-	-	-
7	2	B2	moderate	mild	moderate	mild	neg	-	-	-	-	-
8	3	B2	moderate	mild	mild	moderate	neg	-	-	-	-	-
9	6	B3	moderate	moderate	moderate	severe	neg	-	-	-	-	mild
10	2	Absent	moderate	mild	mild	mild	neg	-	-	-	-	mild
11	6	B3	Severe	moderate	severe	mild	pos **	-	-	-	-	-
12	4	B2	moderate	moderate	moderate	moderate	pos *	-	-	mild cellular	-	-
13	2	Absent	moderate	mild	mild	mild	neg	-	-	-	-	-
14	3	B2	moderate	moderate	moderate	moderate	pos *	-	-	-	-	-
15	4	A	Mild	mild	-	-	neg	-	-	-	-	-
16	2	Absent	Mild	mild	-	mild	neg	-	-	-	-	-
17	/	/	/	/	/	/	/	/	/	/	/	/
18	5	B1	moderate	moderate	mild	mild	pos *	-	-	-	-	mild
19	6	B2	Severe	moderate	moderate	moderate	pos **	mild	mild	-	-	-
20	/	Absent	moderate	mild	moderate	moderate	neg	-	-	-	moderate interstitial	-
21	/	/	/	/	/	/	/	/	/	/	/	/

PVAN: Polyomavirus Associated Nephropathy; Neg: Negative; pos: Positive; * in the tubular basal membrane; ** in interstitial capillaries and glomerular basal membrane.

**Table 6:** Third biopsy.

Patients	DNAemia (Log10)	PVAN	Interstitial Fibrosis	Capillaritis	Tubulitis	Interstitial Inflammatory Infiltrate	C4d	Acute Glomerulopathy	Chronic Glomerulopathy	Acute Rejection	Chronic Rejection	Cytoplasmic Vacuolation of Tubular Epithelium (Acute Toxicity by Calcineurin Inhibitors)
1	4	C	Severe	mild	mild	moderate	neg	-	-	-	-	-
3	2	Absent	Severe	moderate	moderate	moderate	neg	-	-	-	moderate interstitial	-
4	2	Absent	Severe	mild	moderate	moderate	neg	-	-	-	-	-
6	3	Absent	Moderate	mild	mild	moderate	neg	-	-	-	-	-
7	2	Absent	Severe	mild	moderate	moderate	neg	-	-	-	-	-
14	2	B2	Severe	mild	mild	mild	pos *	-	-	-	-	-
18	6	B2	Moderate	moderate	moderate	mild	neg	-	-	-	-	mild
19	6	C	Severe	moderate	moderate	moderate	POS *, **	mild	mild	-	-	-

PVAN: Polyomavirus Associated Nephropathy; Neg: Negative; pos: Positive; * in the tubular basal membrane; ** in interstitial capillaries and glomerular basal membrane.

**Table 7:** Fourth biopsy.

Patients	DNAemia (Log10)	PVAN	Interstitial Fibrosis	Capillaritis	Tubulitis	Interstitial Inflammatory Infiltrate	C4d	Acute Glomerulopathy	Chronic Glomerulopathy	Acute Rejection	Chronic Rejection	Cytoplasmic Vacuolation of Tubular Epithelium (Acute Toxicity by Calcineurin Inhibitors)
1	2	Absent	Severe	moderate	moderate	moderate	neg	-	-	-	-	-
18	4	B2	Moderate	severe	moderate	mild	neg	-	-	-	-	-
19	5	C	Severe	mild	severe	severe	pos *	moderate	moderate	-	-	-

PVAN: Polyomavirus Associated Nephropathy; Neg: Negative; pos: Positive; * in the tubular basal membrane.
